# Investigation of diffusion length distribution on polycrystalline silicon wafers via photoluminescence methods

**DOI:** 10.1038/srep14084

**Published:** 2015-09-14

**Authors:** Shishu Lou, Huishi Zhu, Shaoxu Hu, Chunhua Zhao, Peide Han

**Affiliations:** 1 State Key Laboratory on Integrated Optoelectronics, Institute of Semiconductors, Chinese Academy of Sciences, Beijing 100083, China

## Abstract

Characterization of the diffusion length of solar cells in space has been widely studied using various methods, but few studies have focused on a fast, simple way to obtain the quantified diffusion length distribution on a silicon wafer. In this work, we present two different facile methods of doing this by fitting photoluminescence images taken in two different wavelength ranges or from different sides. These methods, which are based on measuring the ratio of two photoluminescence images, yield absolute values of the diffusion length and are less sensitive to the inhomogeneity of the incident laser beam. A theoretical simulation and experimental demonstration of this method are presented. The diffusion length distributions on a polycrystalline silicon wafer obtained by the two methods show good agreement.

Within the past years, photoluminescence (PL) imaging has evolved to be an important and representative method for measurement of semiconductor devices^1^,^2^. The features of PL imaging are non-contact (without damaging the sample), fast, and on-line which make it prominent from other testing methods. What’s more, it can be used in every step of the whole production process from semiconductor raw materials to the finished photovoltaic devices. Applications of PL imaging method contain characterizing raw material quality (for example, dislocation defects within the material)^3^,^4^, local effective minority carrier lifetime and minority carrier diffusion length^5^,^6^. It can also characterize the leakage positions within the photovoltaic cells, as well as series resistance and doping concentrations of solar cells, etc.

At present, methods of characterizing diffusion length changes in space include light beam induced current (LBIC) imaging^7^,^8^, electroluminescence (EL) imaging^9^ and photoluminescence (PL) imaging. LBIC imaging has the advantage of high precision on the testing data. But it would cost a very long time, such as detecting a 125 × 125 square millimeters solar cell should require several hours. EL imaging significantly shortens the measurement time (it only needs a few seconds). Whereas, applied voltage must be added to the sample under test in this method which will have some bad influence to the sample more or less. At the same time, as it requires the sample under test with at least an electrode, restricts it to be applied to a semiconductor raw material. PL imaging overcomes the above problems. The excess carriers within the semiconductors are stimulated by laser instead of applied voltage. However, the existing studies dedicated to extracting quantitative information on spatially resolved recombination properties of silicon wafers from PL have one common problem. They rely on further measurement techniques in addition to PL like CDI/ILM (carrier density imaging/infrared lifetime mapping), or require the detailed data of the samples under test and test equipment themselves^10^,^11^. Recently some researchers have focused on the study of free carrier absorption and minority carrier distribution in the grain boundaries via PL method^12^. Whereas, they have not showed an accurate and feasible method to investigate the diffusion length distribution yet.

In this work, we present a method of extracting quantified information of the minority carrier diffusion length distribution of a polycrystalline silicon wafer using only basic PL data. To obtain the numerical relationship between the diffusion length and the final PL intensity of each pixel detected by a charge-couple device (CCD), we first study the physical mechanism of the silicon PL effect. The entire process consists of absorption of the laser beam by the silicon, spontaneous radiation within the material, photon reabsorption and transmission inside the wafer and PL signal acquisition by CCDs. From this process, we obtain the direct numerical relationship between the diffusion length (the desired information) and the pixel data on the CCD (the information that can be obtained). However, many other parameters are required to obtain the diffusion length from the data on the CCD, such as the CCD camera’s conversion factor from photoelectrons to digital units *k*_*AD*,_ and the effective solid angle of detection, Ω. Therefore, we also investigate the relationship between PL images taken in different wavelength ranges or from different sides. We find that the ratio of different PL images can also reveal the diffusion length distribution; that is, different values of the ratio correspond to different diffusion lengths. On the basis of this finding, we extract the quantified diffusion length distribution of a polycrystalline silicon wafer by two methods. In the first method, PL images are obtained with and without an 1100 nm long-pass filter. From the ratio of these two images, we can obtain the diffusion length distribution. The second method is consistent in principle with the first, but detects the photoluminescence from the front and rear sides of the wafer. The two methods can be used to verify each other, and they can also be applied to other semiconductor raw materials, which would improve our understanding of specific materials and guide device fabrication.

## Results

### Experimental scheme

**Figure 1:**
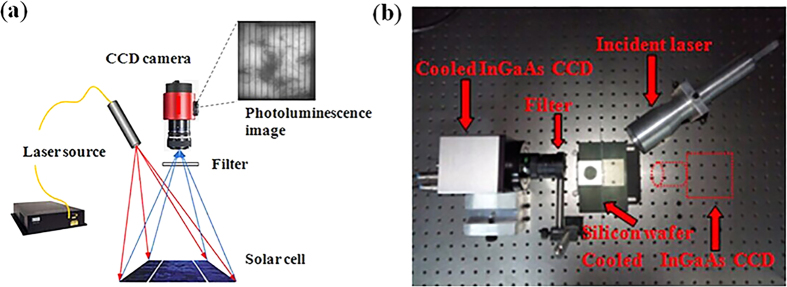
Experimental setup. (**a**) Schematic diagram of the photoluminescence imaging system. (**b**) An exterior view of the experimental system.

A certain wavelength laser is used to irradiate to the surface of a semiconductor, then semiconductor materials absorb the photons and excite electron-hole pairs. The electron-hole pairs will recombine and emit fluorescence in a short period of time. Using the cooled charge coupled device, the image was displayed on the computer. This is the brief principle of PL imaging. Figure 1 shows the schematic diagram of the photoluminescence imaging system used in this work, and the setup details are described in the Method section.

A fiber coupled output semiconductor laser (wavelength of 808 nm/980 nm, 35 W, numerical aperture 0.22) is used for this experiment. The laser beam is homogenized to a 3 × 4 square centimeters beam by an optical system, and then irradiate to the sample. The sample is a 200 μm thickness N-type polycrystalline silicon wafer placed on a stage vertically. A thick surface oxidation layer (about 50 Å) has been formed on the wafer to achieve a certain surface recombination velocity (about 10^4^ cm/s). A cooled InGaAs CCD is used to capture the PL signal of the silicon wafer. An 1100 nm long-pass optical filter is placed in front of the CCD camera for choice. The response curves of InGaAs CCD and filter are shown in the Method section at the end of this paper. As the sensitivity range of the camera is from 900 nm to 1700 nm, by only using a long-pass filter, defect band emissions may contain in the PL images. However, in polycrystalline silicon wafers, the effect of defect band emissions to the whole photoluminescence intensity could be neglected^2^.

### The dependence on the laser power

**Figure 2:**
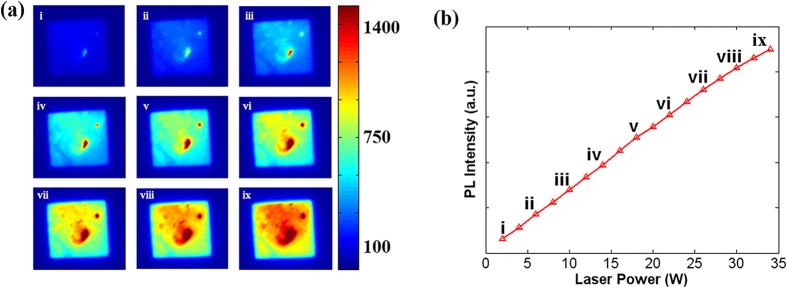
PL intensity variation with laser power. (**a**) Photoluminescence images with different laser power varying from 2 W to 34 W. (**b**) A plot of the integrated PL intensity versus laser power.

To study the effect of the laser power on the PL results, we perform the following experiments. The sample under test is a 200 μm thick polycrystalline silicon wafer. The laser and cooled InGaAs CCD are placed on opposite sides of the sample under test. The output power of the laser is varies from 2 to 34 W in steps of 2 W. All the pixel data in one PL image detected by the InGaAs CCD are added. Figure 2 shows that the light intensity variation curves of the resulting 17 PL images exhibit basically linear variation (because space is limited, we show only 9 pictures on the left corresponding to points from *i* to *ix* in the variation curve). Light saturation does not occur. We can conclude from this result that the laser power in this arrangement causes minority carrier injection in the semiconductor. Consequently, the entire output power of the laser in the available ranges can be used in the following experiments, we choose a relatively high output power to reduce the detection time of the CCD camera.

### Analysis of three different types of polycrystalline silicon wafer

To obtain an accurate diffusion length distribution, we should first investigate the factors that influence the PL intensity. At the same time, the applicability of this PL system to different types of polycrystalline silicon wafers must be studied. Therefore, we choose three types of polycrystalline silicon wafers, which are labeled A, B, and C, for this experiment. They are classified by visual inspection. Both sides of A are exactly the same, with relatively large and uniformly distributed grains; B’s two sides appear totally different, where the grains are relatively small and nonuniformly distributed; C’s two sides appear the same overall, and the grains are also small and nonuniformly distributed.

**Figure 3:**
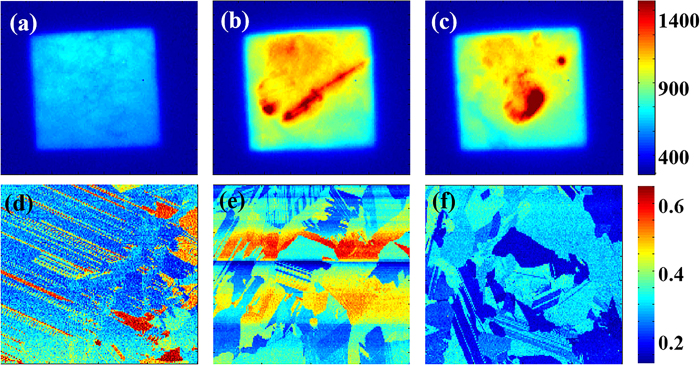
(**a**–**c**) Photoluminescence images of sample A, B, and C. (**d**–**f**) Reflectivity scanning images of sample A, B, and C.

Figure 3(a–c) show PL images of these three polycrystalline silicon wafers, where the incident laser has an 808 nm wavelength. The PL intensities of the three wafers detected by the InGaAs CCD differ greatly. The sums of the gray values of each pixel in the three images are 3.86×10^6^, 4.69×10^6^, 5.18×10^6^ respectively. However, we could not conclude from the data that B is the best wafer as it produced more photons, indicating fewer defects and a longer diffusion length. The PL intensity is known to depend mainly on the properties of the interior of the semiconductor itself. However, it would also be influenced by other factors such as the homogeneity of the laser beam, and differences in the reflectivity at different points on the silicon wafer surface. A laser beam homogeneity of up to 90% can be confirmed, and the three wafers are tested by the same homogeneous system. Thus, the laser beam homogeneity would not affect this comparative analysis of the light intensity. However, the reflectivity distribution is expected to be problematic, so we then tested the three wafers using point-by-point reflectivity scanning (the details are discussed in the Method section). As shown in Fig. 3(d–f), these three samples exhibit different reflectivity properties. The average reflectivities of them are 0.45, 0.31, and 0.24, respectively. If we add the effect of the reflectivity to the description of the above three PL images, we could obtain a totally different result. The actual PL intensities of these three wafers are ordered as follows: A > C > B. Considering the features of these three types of wafers, A is most likely to have the highest PL intensity, as it has relatively large and uniformly distributed grains. To confirm this, we made four-point-probe resistivity measurements. The square resistances of them are 63Ω/◽, 83Ω/◽, and 76Ω/◽. The square resistance is inversely proportional to the doping concentration *N*_*A*_*/*_*D*,_ so samples with smaller square resistance values have a larger doping concentration and should produce more spontaneously emitted photons. Samples with smaller square resistance values (such as sample A) lead to have higher relative PL intensities. This is also explained later in this paper near equations (2) and (3) where radiative recombination is a function of carrier concentration or doping. So, radiative recombination is expected to be larger for more heavily doped samples (or less resistivity). This can explain why sample A has the highest PL intensity among these three samples.

From the analysis of the above results, we can confirm the accuracy of this PL imaging method. PL imaging of the wafers provide information on the carrier density distribution and in principle reveals the effect of the minority carrier diffusion length distribution. Finally, we choose sample B for a further study on the determination of the quantified diffusion length distribution.

### Determination of the diffusion length of a silicon wafer

**Figure 4:**
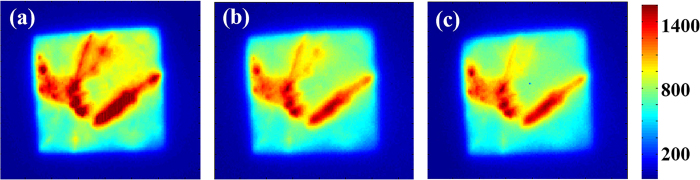
Photoluminescence images of sample B with (a) no filter from rear side, (b) an 1100 nm long pass filter from rear side and (c) an 1100 nm long pass filter from front side.

On the basis of the above preparations, we took PL images at the same position on sample B from the front side (with an 1100 nm long-pass optical filter) and rear side (with or without the filter). For the front side images, the side incident laser and InGaAs CCD camera are on the same side, whereas for the rear side images, they are on opposite sides. The PL images are shown in Fig. 4.

**Figure 5:**
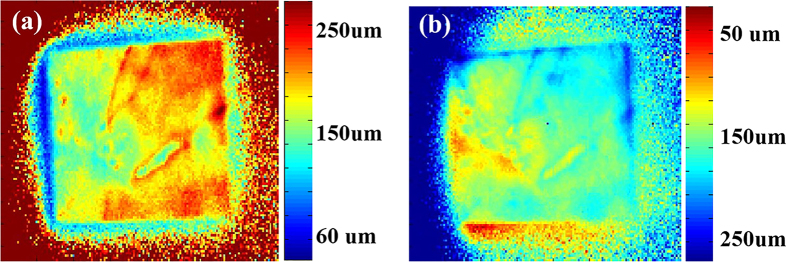
Minority carrier diffusion length distribution images of sample B with (a) the first method and (b) the second method.

Two methods are used to fit the diffusion length distribution mapping of the silicon wafer. One compares the wavelength variation tendencies of the PL intensity with and without the filter. The other compares the photon transmission toward the front and rear surfaces. The theory of the two methods is discussed in detail in the Discussion section. The results are shown in Fig. 5.

Figure 5(a) shows the minority carrier diffusion length distribution mapping using the filter method (first method), where red indicates a longer diffusion length, and blue represents a shorter diffusion length. The mapping was made using the ratio of the PL intensities in images of the rear side (with and without an 1100 nm long-pass filter). Figure 5(b) also shows the minority carrier diffusion length distribution mapping, but it was obtained using the second method, where blue represents longer diffusion lengths, in contrast to the results of the first method. It was obtained using the ratio of the PL intensities in images of the front and rear sides of the wafer with an 1100 nm long-pass filter. The results of the two methods agree well overall. However, we find that the result of the second method does not look as distinct as that of the first one, and there may be local regions that cannot be used. This is due mainly to the slight position shift between images of the front and rear sides, which would cause incomplete overlap between these two PL images. However, this will not critically influence the characterization of the minority carrier diffusion length.

## Discussion

The imaging theory of the quantized diffusion length distribution is based on the fundamental nature of the semiconductor itself. Therefore, we should investigate the entire process, starting with the production of extra carriers and proceeding through detection of PL photons by the CCD. As we know, the laser as the incident source irradiates to the surface of the polycrystalline silicon wafer, and light absorption occurs in a thin surface layer with a thickness comparable to the diffusion length. The continuity equation of the excess minority carrier density can be written as equation (1).




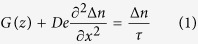




Where, *G* is the body excess carrier generation rate, *D*_*e*_ is the diffusion coefficient of electrons. The photo-generated carriers would then diffuse and recombine in the material. Surface recombination would also occur. Thus, the boundary conditions should be added to the continuity equation, as shown in equation (2).




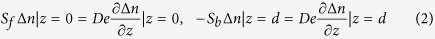




**Figure 6:**
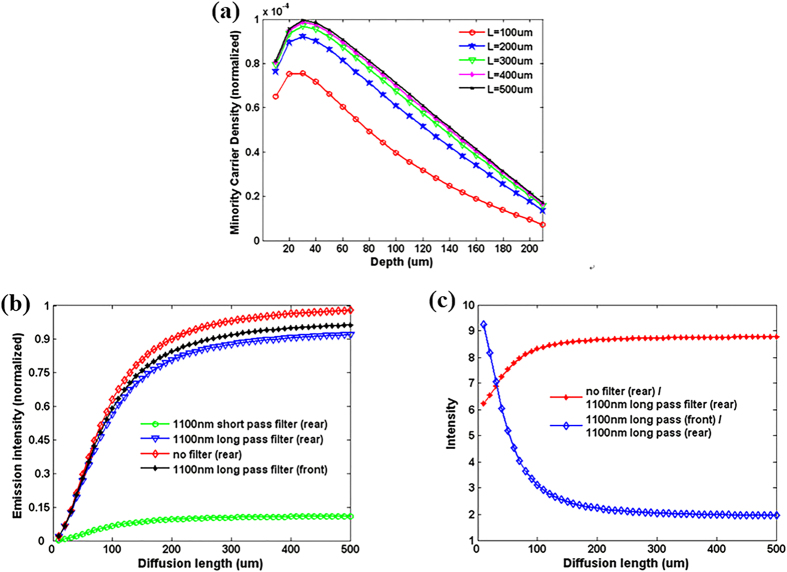
(**a**) Relationship between minority carrier density, depth and diffusion length. (**b**) Numerically integrated detectable photon currents for different cutoff filters. (**c**) Theoretical results for the ratio of the expected luminescence signals as a function of the diffusion length of L.

Figure 6(a) shows the relationship between the minority carrier density and the depth, as well as the relationship between the minority carrier density and the diffusion length. The primary carrier recombination modes in silicon are Shockley-Read-Hall recombination and Auger recombination, whereas the fluorescence PL is caused by spontaneous radiation recombination. According to a generalization of Planck’s law of radiation for non-black bodies, a semiconductor’s rate of spontaneous photoemission per photon energy and volume can be described in terms of its doping concentration *N*_*a*_*/*_*d*_ and an intrinsic equilibrium concentration of excess charge carriers *n*_*i*_. With the absorption coefficient at a specific photon energy and the phase velocity of light in the semiconductor *r*_*sp*_(*Eγ*)^13^, *r*_*sp*_(*Eγ*) can be written as equation (3).




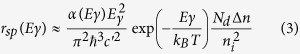




Before the photons produced by spontaneous radiation recombination reach the surface of the silicon wafer, recombination and reflection would occur at the inner surface^14^. Then the emitted photon current measured outside of the sample, *I*_*j*_(*E*_*γ*_) can be expressed as




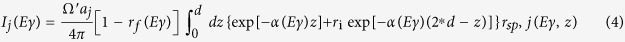







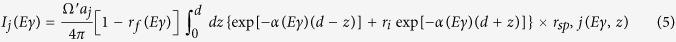




In equations (4) and (5), *d* is the wafer thickness. In the above treatment, it is assumed that nonradiative recombination is the dominant recombination mechanism. The generation of electron-hole pairs by the reabsorption of spontaneously emitted photons is therefore neglected in the continuity equation for the electrons in equation (3). Multiple reflections in the semiconductor are also neglected. To properly model the intensity detected by the CCD camera, Φ_*j*_, the detector’s quantum efficiency *Q*_*cam*_*(E*_*r*_) and the transmission of the long pass filter T_filter_ must be included.




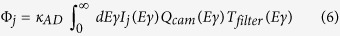




Figure 6(b) shows the numerically integrated detectable photon currents for different cutoff filters and without a filter. The data are normalized at a long diffusion length. The variation as a function of the diffusion length corresponding to the normalized luminescence signal depends on the filter that is used. For an 1100 nm short-pass filter, a strong dependence of the normalized luminescence signal on the diffusion length *L* is predicted for *L* < 100 μm and relatively little additional variation is predicted for *L* > 100 μm. This can be easily explained by the fact that the additional carriers that diffuse deeper into the wafer for longer diffusion lengths do not affect the measured signal at short wavelengths because of reabsorption. However, for the long-pass filter and without a filter, the photons with long penetration depths are measured. In that case, the variation in the normalized luminescence signal is stronger for long wavelengths. Therefore, we found that the results of the short-pass filter give more information about the carrier density near the wafer surface, whereas those measured without a filter or with a long-pass filter reveal more information about the entire wafer.

It may appear that we could just use the relationship between the measured luminescence intensity and the diffusion length from the above expressions to determine the diffusion length distribution. We do not do that for the following two reasons. First, it is necessary to calibrate the intensity curve by comparison with the intensity of a standard solar cell or wafer with a known diffusion length. Second, by using multiple PL images, the effect of laser beam inhomogeneity on the diffusion length distribution can be removed.

We proposed a method that eliminates both of these problems by measuring at least two PL images taken in different wavelength ranges or from opposite sides. Figure 6(c) shows the theoretical results for the ratio of the expected luminescence signals as a function of the diffusion length *L*. The ratio of two relative luminescence signals measured in different ways gives a number that indicates the absolute diffusion length. From the above results, we find that the luminescence intensities do not vary significantly when *L* > 200 μm and S = 10^4^ cm/s. The surface recombination velocity is a key element in our proposed model. A deviation in the surface recombination velocity would lead to errors in the final calculated diffusion length. Therefore, we set up the surface recombination velocity in the model the same as the sample to have better fitting. In this case, the analysis of the luminescence intensity ratios yields an effective diffusion length instead of the bulk diffusion length. Therefore, we could determine the numerical value of the diffusion length distribution using this method.

**Figure 7:**
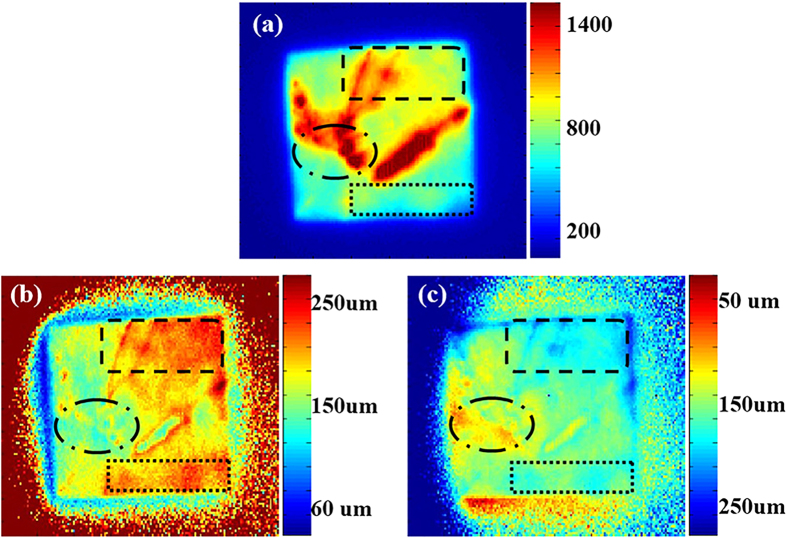
(**a**) Photoluminescence image of sample B. (**b**) Numerical value of the diffusion length distribution of sample B with first method. (**c**) Numerical value of the diffusion length distribution of sample B with second method.

Figure 7(b,c) show the numerical value of the diffusion length distribution of sample B obtained by the above method. Figure 7(b) is made by fitting the results for two different wavelength ranges, those in PL images taken without a filter and with an 1100 nm long-pass filter. Figure 7(c) is made by fitting the results for opposite sides, those from PL images detected from the front and rear sides of the silicon wafer. These two pictures agree well overall. As shown in Fig. 6(c), the intensity ratio of PL images at two different wavelengths (red line) changes very little when the diffusion length exceeds 250 μm.

Therefore, in the conversion of the intensity ratios into the diffusion length, all the areas with intensity ratios greater than 8.5 were converted into a diffusion length longer than 250 μm. The same mean was also used in the method using PL images of opposite sides [blue line in Fig. 6(c)], although the correspondence between the color and diffusion length values is exactly opposite to that in the first method. For comparison, Fig. 7(a) shows a map of the PL image taken without a filter from the rear side. We choose three different positions on the wafer for comparison with the diffusion length distribution results we obtained. The area enclosed by the rounded square in Fig. 7(a) (top right corner) shows a relatively high PL intensity, and in Fig. 7(b,c), the areas are also regions of longer diffusion length. The area enclosed by an oval in Fig. 7(a) shows a high PL intensity, whereas the same region in Fig. 7(b,c) shows a shorter diffusion length. A possible explanation is that this area may contain grain boundaries, which can radiate light at other wavelengths that contributes to the PL intensity. However, they are actually not areas of long diffusion length. The last one we choose to compare is the area enclosed by a square in Fig. 7(a) (bottom right corner). Because of incident laser beam inhomogeneity, that area shows low PL intensity, although it is a region of long diffusion length, as shown in Fig. 7(b,c).

**Figure 8:**
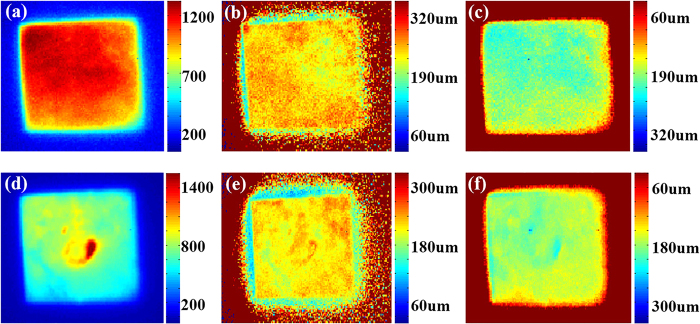
(**a**,**d**) Photoluminescence images of sample A and sample C. (**b**,**e**) Numerical values of the diffusion length distribution of sample A and sample C with first method. (**c**,**f**) Numerical values of the diffusion length distribution of sample A and sample C with second method.

Sample A and sample C were also tested using the same methods we proposed. The results of diffusion length distribution of them are shown in Fig. 8. That could be a verification of our methods. The experimental results of sample A and C are not as well as the one of sample B. One reason is that the diffusion lengths of sample A and C are longer than sample B, which may cause some error in the long diffusion length area. The other reason is that the surface recombination velocities of these three samples would have some deviations more or less which will lead to some errors using the same simulation model.

To summarize, from the above analysis and comparison, we conclude that the methods we proposed to quantitatively determine the spatially resolved diffusion length are accurate and feasible. Furthermore, they can also eliminate the effects of laser beam inhomogeneity and some other characteristics of the wafer itself on the resulting diffusion length distribution. However, these methods also have some limitations. The variation in the PL intensity ratio with the diffusion length would vary with the surface recombination velocity. Therefore, the surface recombination velocity of each type of sample should be known and recalculated in the model. As the variation curve of the PL intensity ratio is weak for long diffusion lengths, this method become less sensitive to diffusion lengths that are comparable to or larger than the wafer thickness. In addition, surface texturing or nanostructures in the samples under test may cause errors because they affect the internal light path.

## Conclusion

We proposed two methods of quantitatively determining the spatially resolved diffusion length. One method is to measure two PL images taken with and without an 1100 nm pass filter from the rear side of wafer. The other method is to measure two PL images taken from the front and rear sides of the wafer. The two methods were discussed theoretically and demonstrated experimentally on a polycrystalline silicon wafer. Three different features of samples under test were tested using these methods. Good agreement was observed between these two methods. These methods can be applied to silicon wafers at all stages of solar cell production, from raw wafers through to finished solar cells. They can also eliminate the effects of laser beam inhomogeneity and some other characteristics of the wafer itself on the resulting diffusion length distribution.

## Method

### Laser beam homogenizing system design for photoluminescence

Based on waveguide coupling theory, we put forward a scheme for large area uniform laser irradiation, and prove it by theoretical simulation and experiment. The incident semiconductor laser beam is shaped with a kaleidoscope which can change the uneven intensity distributed multimode laser beam into a flat distributed uniform spot. And then double telecentric and 10 × zoom eyepiece lens are respectively added after the kaleidoscope to image and amplify the shaped uniform beam onto the irradiation plane. The simulation results of overall light intensity uniformity are both greater than 89% under the irradiation area of 3.5 × 3 square centimeters and 10 × 10 square centimeters. In the practical application, it is also greater than 85%. Finally, we apply this optical system (double telecentric) in the semiconductor photoluminescence imaging detection, the light homogenized effect is ideal^15^.

### Reflectivity scanning imaging system

**Figure 9:**
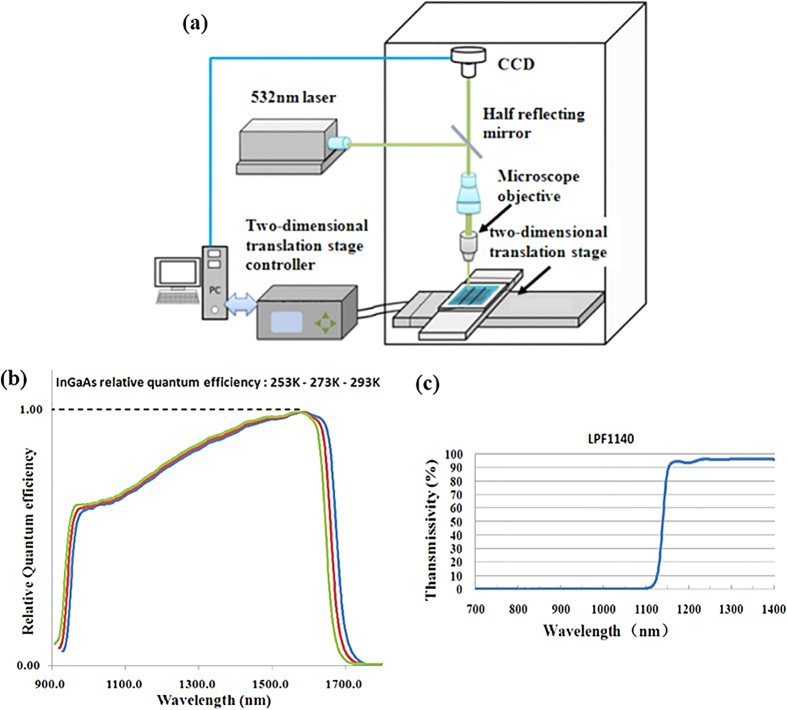
(**a**) Schematic diagram of the reflectivity scanning imaging system. (**b**) Wavelength response curve of cooled InGaAs CCD. (**c**) Transmission curve of the 1100 nm long pass filter.

A 200 mW, 532 nm wavelength laser is used as the incident light source of the reflectivity scanning imaging system. Before the laser beam irradiates to the surface of sample under test, it is reflected by a half transparent and half reflecting mirror, then focused by a microscope objective. The final diameter of laser beam on the sample under test is about 100 μm. A CCD camera is placed over the half mirror to detect the reflective light from the sample surface. The sample under test is placed on a two-dimensional translation stage. By moving the two-dimensional translation stage, reflectivity scanning image of the sample could be achieved. In order to obtain the absolute value of reflectivity, we use a standard sample to calibrate. The reflectivity scanning images of A, B, and C, in this paper are obtained by this method. Schematic diagram of the reflectivity scanning imaging system is shown in Fig. 9(a).

### Response curve of InGaAs CCD and filter

The light response data of cooled InGaAs CCD camera (Xeva 1.7 320 TE3 camera from sInfraRed company) and transmission data of 1100 nm long-pass filter (from Mega-9 company) is used in this paper for calculation of diffusion length. The response curves of them are obtained from their manufacture factory [shown in Fig. 9(b,c)].

## Additional Information

**How to cite this article**: Lou, S. *et al.* Investigation of diffusion length distribution on polycrystalline silicon wafers via photoluminescence methods. *Sci. Rep.* **5**, 14084; doi: 10.1038/srep14084 (2015).
